# On Reactions with the Newer Antibiotics

**Published:** 1952-04

**Authors:** P. G. Dalgleish

**Affiliations:** Registrar, Department of Medicine, University of Bristol


### ON REACTIONS WITH THE NEWER ANTIBIOTICS

BY

P. G. DALGLEISH, M.D., M.R.C.P.
*Registrar, Department of Medicine, University of Bristol*

The newer antibiotics, chloramphenicol and aureomycin, produce side effects three or four times more frequently than does penicillin. Fortunately most are not serious. Reactions are more likely to happen when the daily dosage is high or when administration is prolonged. Most of the untoward effects occur in the gastro-intestinal tract and this system seems more susceptible in women than in men. Other systems of the body are affected far less frequently.

With oral therapy about forty per cent of patients develop some symptoms or signs in the mouth. They complain of dryness, cracks at the corners of the mouth, or soreness of the tongue; and they may complain of an unpleasant taste in the mouth or lose the sense of taste altogether. The changes that are seen are varied. Most often the tongue is red and smooth and there is loss of the filiform papillae. Less frequently there is a hypertrophic glossitis and, when the papillae become covered with fungus, there may be a true black tongue (Tomaszewski, 1951). Other changes seen are vesicles and papules on the palate. It is not certain why these changes occur but there are two possibilities—first, as the changes resemble riboflavin deficiency (Harris, 1950), it was thought that the antibiotics somehow brought them about by interfering with vitamin B synthesis in the bowel. This seems unlikely because riboflavin does not prevent their occurrence though it hastens their disappearance. The other more likely possibility is that the normal bacterial flora in the mouth are killed off by the antibiotics and that Yeasts and Monilia (*Candida albicans*) grow there without competition.

Some form of indigestion is frequent; this amounts to flatulence in twenty-five per cent of cases and nausea in twenty per cent but in five per cent there is actual vomiting (Tomaszewski, 1951). The flatulence may be severe and may cause epigastric pain. These symptoms are lessened without interfering with their action by giving the drugs with milk or food. Antacids and alkalis should not be used to relieve the dyspepsia as they reduce the activity of the drugs which are only stable in an acid medium (Harvey *et al.*, 1949). The appetite varies from patient to patient and cravings may be present as in pregnancy.

In the rectum and anus side effects have been reported in up to fifty per cent of cases. The stools often become large, loose and odourless soon after treatment begins. Bacteriological examination of the faeces shows that the *B. coli* have been largely replaced by Yeasts and Monilia. This latter organism probably accounts for the troublesome proctitis that occurs in ten to twenty per cent of cases (Harris, 1950). The perianal skin becomes red and thickened and whitish plaques and fissures may be seen. Genital pruritus is often present, particularly in women in whom there may be a vulvo-vaginitis due to Monilia (Nayfield, 1951; Reiches *et al.*, 1951). Antihistamine compounds give only partial relief from the pruritus though oestrogens may relieve the vulvo-vaginitis (Harris, 1950). Aureomycin tends to cause this group of symptoms more readily than does chloramphenicol.

In the same way that these drugs destroy the normal bowel flora and allow Monilia to flourish so they may permit pulmonary moniliasis to occur when used for the treatment of chronic lung infections. The possibility of a disseminated mycosis occurring, similar to that which has been described during penicillin therapy, should be borne in mind.

No harmful effects on the kidneys have yet been reported.

It has been reported that in the presence of already existing liver damage heavy doses of aureomycin may increase the severity of the damage (Lepper *et al.*, 1951). On the other hand, aureomycin is said to have had good effect in the treatment of patients with chronic liver disease (Shaffer *et al.*, 1950).

Granulocytopenia has occurred during chloramphenicol therapy though it was not severe and was readily reversible (Gill, 1950; Volini *et al.*, 1950). A fatal case of aplastic anaemia while on chloramphenicol is on record (Rich *et al.*, 1950) and the author knows of another in this country. No other definite effects on the blood have yet been proved though much work on this subject is in progress. Aureomycin has recently been reported to be effective in the treatment of pernicious anaemia (Lichtman *et al.*, 1950) and in the anaemia of the " intestinal loop " syndrome in rats (Watson *et al.*, 1952). This drug has also been found to increase growth when given to young animals (*Lancet*, 1950) and possibly children too (Robinson, 1952).

Aureomycin in heavy dosage may produce euphoria, drowsiness, irritability and mental confusion, and a possible case of polyneuritis has been reported (Marten, 1951). Chloramphenicol sometimes produces weakness of accommodation and general muscular weakness (Gray, 1950). All these effects are transient.

Either drug may produce a fleeting local or general urticaria during the first or subsequent courses. Erythema and vesiculo-papular eruptions sometimes appear particularly on the dorsum of the hands and about the face. They may be precipitated by exposure to sunlight and are said to occur more frequently in those who take alcohol. These rashes are considered by some to be due to sensitization of the patient to the drugs (Peck *et al.*, 1950; Bedford, 1951).

Intramuscular injections of aureomycin and terramycin are painful and cause much tissue reaction so they are rarely given this way. Intravenous injections are liable to cause phlebitis unless given well diluted. Chloramphenicol has, so far, been given by mouth only. In typhoid fever this drug may cause vasomotor collapse and an abrupt fall of temperature during the first forty-eight hours of treatment (*Brit. Med. J.*, 1950). The mechanism of this is not clear.

At the time of writing reports on side effects from terramycin are scanty though this drug has been in use in the United States for nearly two years. They appear to be similar to those produced by aureomycin.

### SUMMARY

Some of the side effects of chloramphenicol and aureomycin are described. They fall into two major groups: those on the gastro-intestinal tract which are common, and those on the other systems of the body which are uncommon.

The former, though troublesome, are not dangerous and do not often necessitate stopping treatment.

### REFERENCES

Bedford, P. D. (1951). *Brit. Med. J.*, **1**, 1428.
*Brit. Med. J.* Annotation (1950). Ibid., **1**, 829.
Gill, P. F. (1950). *Med. J. Australia*, **1**, 768.
Gray, J. D. (1950). *Lancet*, **1**, 150.
Harris, H. J. (1950). *J. Amer. Med. Ass.*, **142**, 161.
Harvey, J. L., Mirick, G. S., Schaub, I. G. (1949). *J. Clin. Invest.*, **28**, 987.
*Lancet* Annotation. (1950). *Lancet*, **1**, 912.
Lepper, M. H., *et al.* (1951). *Arch. Int. Med.*, **88**, 271.
Lichtman, H., Ginsberg, V., Watson J. (1950). *Proc. Soc. Exp. Biol. and Med.* **74**, 884.
Marten, R. H. (1951). *Lancet*, **2**, 1143.
Nayfield, R. C., (1951). *Amer. J. Obst. and Gyn.*, **62**, 452.
Peck, S. M., and Feldman, F. F. (1950). *J. Amer. Med. Ass.*, **142**, 1137.
Reiches, A. J. and Webb, P. K. (1951). *Arch. Derm. and Syph.*, **64**, 63.
Rich, M. L., Ritterhoff, R. J., Hoffmann, R. J. (1950). *Ann. Int. Med.*, **33**, 1459.
Robinson, P. (1952). *Lancet*, **1**, 52.
Shaffer, J. M., *et al.* (1950). *Amer. J. Med. Sci.*, 220, 1.
Tomaszewski, T. (1951). *Brit. Med. J.*, **1**, 388.
Watson, G. M., Witts L. J. (1952). *Brit. Med. J.*, **1**, 13.
Volini, I. F., *et al.* (1950). *J. Amer. Med. Ass.*, **142**, 1333.

